# Prednisolone versus placebo addition in the treatment of patients with recent-onset psychotic disorder: a trial design

**DOI:** 10.1186/s13063-020-04365-4

**Published:** 2020-06-08

**Authors:** Lyliana G. Nasib, Iris E. Sommer, Inge Winter - van Rossum, Jacqueline de Vries, Shiral S. Gangadin, Priscilla P. Oomen, Gurmeet Judge, Renske E. Blom, Jurjen J. Luykx, Nico J. M. van Beveren, Natalie D. Veen, Rune A. Kroken, Erik L. Johnsen

**Affiliations:** 1 Department of Psychiatry, UMC Brain Center, University Medical Center Utrecht, Utrecht University, Utrecht, The Netherlands; 2 Cognitive Neurosciences, Department of Biomedical Sciences of Cells & Systems, University Medical Center Groningen, Groningen, The Netherlands; 3 Department of Biological and Medical Psychology, University of Bergen, Bergen, Norway; 4 Reinier van Arkel, ‘s-Hertogenbosch, The Netherlands; 5 GGZ Delfland, Delft, The Netherlands; 6 Department of Translational Neuroscience, UMC Brain Center, University Medical Center Utrecht, Utrecht University, Utrecht, The Netherlands; 7 Department of Psychiatry, ZNA Hospitals, Antwerp, Belgium; 8 Department of Psychiatry, SymforaMeander Hospital, Amersfoort, The Netherlands; 9 Antes Center for Mental Health Care, Rotterdam, The Netherlands; 10 Department of Psychiatry, Erasmus MC, Rotterdam, The Netherlands; 11 Department of Neuroscience, Erasmus MC, Rotterdam, The Netherlands; 12 Norment, Division of Psychiatry, Haukeland University Hospital, Jonas Lies vei 65, 5021 Bergen, Norway; 13 Department of Clinical Medicine, University of Bergen, Pb 7800, 5020 Bergen, Norway

**Keywords:** Neuro-inflammation, Psychotic disorders, Prednisolone, Treatment, MRI

## Abstract

**Background:**

The symptom severity of a substantial group of schizophrenia patients (30–40%) does not improve through pharmacotherapy with antipsychotic medication, indicating a clear need for new treatment options to improve schizophrenia outcome. Meta-analyses, genetic studies, randomized controlled trials, and post-mortem studies suggest that immune dysregulation plays a role in the pathophysiology of schizophrenia. Some anti-inflammatory drugs have shown beneficial effects on the symptom severity of schizophrenia patients. Corticosteroids are effective in various chronic inflammatory and autoimmune disorders. Prednisolone, a potent glucocorticosteroid, has minor mineral-corticosteroid potencies and can adequately pass the blood–brain barrier and its side effects and safety profile are well known. Therefore, the effect of prednisolone can be studied as a proof of concept for immune modulation as a treatment for schizophrenia.

**Methods/design:**

In total, 90 subjects aged 18–70 years and diagnosed with schizophrenia, schizoaffective disorder, or schizophreniform disorder (Diagnostic and Statistical Manual of Mental Disorders-IV (DSM-IV) 295.x) or psychosis not otherwise specified (NOS; 298.9) will be included. The time interval between the onset of psychosis and study entry should not exceed 7 years. Patients will be randomized 1:1 to either prednisolone or placebo daily for a period of 6 weeks in addition to a stable dose of antipsychotic medication. Study medication will be initiated at 40 mg for 3 days, after which it will be tapered down within 6 weeks after initiation, following inflammatory bowel diseases treatment guidelines. Primary outcome is change in symptom severity, expressed as change in total score on the Positive and Negative Symptom Scale (PANSS) from baseline to end of treatment. Cognitive functioning (measured through the Brief Assessment of Cognition in Schizophrenia (BACS)) and change in Global Assessment Functioning (GAF) and depressive symptoms as measured with the Calgary Depression Scale for Schizophrenia (CDS) will be assessed, in addition to various immunological biomarkers. Secondary outcomes are a 4- and 6-month follow-up assessment of PANSS, BACS, and GAF scores and immunological biomarkers. Additionally, a subgroup of patients will be included in the magnetic resonance imaging (MRI) part of the study where MR spectroscopy and structural, functional, and diffusion MRI will be conducted.

**Discussion:**

It is expected that prednisolone addition to current antipsychotic medication use will reduce symptom severity and will improve cognition when compared to placebo.

**Trial registration:**

ClinicalTrials.gov, NCT02949232 and NCT03340909. Registered 31 October 2016 and 14 November 2017. EudraCT-number 2014–000520-14 and 2017–000163-32.

## Background

Schizophrenia is a severe mental disorder with a worldwide prevalence of around 1%, placing significant burden on global health [1]. The introduction of antipsychotic medications has improved the positive psychotic symptoms of schizophrenia patients [2]. However, schizophrenia remains a severe illness with high morbidity and mortality rates [3]. Despite antipsychotic treatment, negative symptoms seem especially persistent over time, which negatively influences quality of life (QoL) [4].

Cognitive deficits are another factor underlying poor QoL. Although not included in the diagnostic criteria for schizophrenia, cognitive deficits are considered a core feature of schizophrenia symptomatology [5]. Cognition may also be a good predictor of long-term functional outcome [6, 7]. A meta-analysis by Nielsen et al. studying the effect of second generation antipsychotic medication on cognition showed some trend level effects; however, none of the antipsychotic drugs showed a positive uniform effect on cognition [8]. For this reason there is a definite need to improve pharmacotherapy in order to reduce symptom severity and counteract the cognitive decline as well.

The accumulating evidence, derived from genetic, post-mortem, epidemiological, and clinical studies, supporting the role of immune dysregulation in the pathogenesis of schizophrenia may provide an opportunity to expand pharmacotherapeutic options. First, a significant association with the major histocompatibility complex (MHC) region, best known for its role in immunity, on chromosome 6p21.3–22.1 was observed in a large genome-wide association study (GWAS), including over 30,000 patients with schizophrenia [9]. An association between MHC and psychosis was also described by Saito and colleagues [10]. Next to this, increased expression of complement 4 (C4), a protein originating from the MHC region, has been found to be associated with schizophrenia. C4 plays a crucial role in the innate immune system by swiftly identifying and eliminating pathogens [11].

Second, epidemiological studies support the theory that the immune system is involved in schizophrenia. A recent review by Jeppesen and Benros described the epidemiological evidence on bidirectional associations between autoimmune disorders and psychotic disorders [12]. Patients with autoimmune disorders were found to carry an increased risk to develop a psychotic disorder and vice versa, suggesting that similar mechanisms are involved in both disorders. Potential mechanisms and risk factors found in both disorders are the presence of neuronal surface antibodies, dysregulation of T and B cells, and infections (viral or bacterial).

Third, microglia, the immune cells in the central nervous system (CNS), have been suggested to play an important role in neurological function and cognition [13]. A recent meta-analysis on post-mortem brain studies indicated that the number of activated microglia cells in the brains of patients with schizophrenia is significantly increased [14]. Microglia can be activated by a systemic inflammatory trigger or by neurodegeneration, aging, and stress [15]. Activated microglia cells can produce free radicals, pro-inflammatory cytokines, and neurotoxic substances, which leads to cell death [16]. The activation of microglia cells provides a possible route by which an inflammatory state in the brain could cause increased grey matter (GM) loss and consequently contribute to more severe negative and cognitive symptoms. However, another meta-analysis found lower levels of glial cell markers in first-episode psychosis and schizophrenia patients in comparison with healthy controls, suggesting a lower density of immune cells and glial cells [17].

Fourth, immune dysregulation in psychotic disorder patients might be reflected by abnormal levels of cytokines and the presence of auto-antibodies in serum and cerebrospinal fluid (CSF) [18]. A recent meta-analysis reported significant increase in interleukin (IL)-6 and IL-8, cytokines involved in inflammatory reactions, in schizophrenia patients compared to healthy controls [19]. A recent study found that symptom severity measured with the Positive and Negative Syndrome Scale (PANSS) [20] was strongly correlated with cytokine levels in the blood that are involved in inflammation [21]. It was found that symptom severity was positively associated with the levels of pro-inflammatory markers such as IL-2R, IL-6, and IL-8. Additionally, a large longitudinal study performed in Finland found an association between high-level C-reactive protein (CRP) values and the development of schizophrenia [22]. Associations between CRP levels and cognition in schizophrenia patients have also been described in a recent systematic review [23]; higher CRP levels (> 3 mg/L) were associated with declined cognitive performance (general intellectual ability, abstract reasoning, working memory, semantic memory, learning abilities, attention, mental flexibility, and processing speed).

Altogether, there is accumulating evidence on the presence of immune dysregulation in patients with schizophrenia, justifying the research into augmenting antipsychotic therapies with anti-inflammatory agents. However, at this stage it is unclear whether an increased pro-inflammatory status is characteristic for all patients with schizophrenia; instead it might be applicable to a specific subset of schizophrenia patients. This aligns with the findings of Schwarz and colleagues, who studied molecular abnormalities in patients with schizophrenia through multiplex immunoassay analyses and were able to distinguish two subgroups based on predominant abnormalities in immune molecules or growth factors and hormones [24].

Immune dysregulation in (a subset of) schizophrenia patients may also result in structural changes in the brain, which could be detected with MRI [25, 26]. These brain changes are possibly present in the earlier stages of the disease [27]. For instance, results from a large meta-analysis suggest that the initiation of white and grey matter (WM and GM) loss is seen in schizophrenia patients before the first psychotic episode [28]. Moreover, the GM and WM loss progresses over the course of the illness. In several studies, GM loss in the first 5 years after diagnosis is associated with poor cognitive functioning and bad outcome [29]. If immune modulation can reduce loss of the GM, it would be expected to be most effective in the first years of illness. Subsequently, both symptom severity and long-term outcome could be improved. However, the available immune studies have focused mainly on chronically ill patients with psychosis [30]. It is important, therefore, to investigate the efficacy of administering a broad-acting, potent immune suppressive agent, especially early in the course of the disease as this may prevent neuronal damage caused by low-grade inflammatory processes in the brain.

Anti-inflammatory drugs that could be used to treat inflammation in psychotic disorder patients include glucocorticosteroids and non-steroidal anti-inflammatory drugs (NSAIDs). Broberg and colleagues studied the risk of developing schizophrenia after glucocorticosteroid use in a large Danish population and found that glucocorticosteroid use is associated with an increased risk of developing schizophrenia [31]. The association might be confounded by the severity of the underlying inflammatory condition, however, as more severe inflammation may be expected to require more potent anti-inflammatory interventions. Further, it was described that short-term treatment with glucocorticosteroids might be effective in treating psychotic episodes, especially in patient subgroups in which increased inflammation is a likely cause of the psychotic symptoms. A meta-analysis by Çakici and colleagues contained 56 randomized clinical trials (RCTs) in which agents with anti-inflammatory potential were studied in patients with schizophrenia [32]. Several anti-inflammatory agents were found to significantly improve schizophrenia symptoms with medium to large effect sizes; aspirin (ES = 0.3), estrogens (ES = 0.78), minocycline (ES = 0.4), and N-acetylcysteine (NAC; ES = 1.0). Some agents were found to have a beneficial effect on cognition: minocycline (attention, executive functioning, and memory), davunetide (verbal learning and memory), and NAC (attention, memory, and executive functioning). The heterogeneity of the cognitive tests used in the study made it impossible to create a quantitative review. Furthermore, the patient samples were heterogeneous, with duration of illness as a potential moderator. It was concluded, therefore, that the effect of immune modulation may be largest in patients with recent onset schizophrenia, as the hypothesized pro-inflammatory status in the brain may be most pronounced during and shortly after disease onset [32].

Although the indications of immune involvement in schizophrenia are accumulating, it is still not possible to reliably specify the exact components of the immune system involved [33]. This leads to uncertainty in the choice of targets and treatment options. Corticosteroids are potent immune modulating agents that target many different aspects of both the innate and the adaptive immune responses. So far, no data exist on the effectiveness of corticosteroids in symptomatology and cognitive deficits in schizophrenia. However, a positive effect on cognition was demonstrated in patients with multiple sclerosis (MS) [34, 35], suggesting that corticosteroids may prevent cognitive deterioration in diseases with a neuroinflammatory component.

Prednisolone is a broad spectrum glucocorticosteroid which interferes with almost all primary and secondary immune cells, including monocytes, microglia cells, T cells, and granulocytes [36]. Furthermore, in contrast to most glucocorticosteroids, prednisolone can easily pass the blood–brain barrier, which is a prerequisite to induce immune modulation in the brain. Finally, there is ample clinical experience with prednisolone and its side effect and safety profiles are well known, as it has been administered for short-term and chronic use in several forms in various conditions for decades [37]. Therefore, treatment with prednisolone can be used as a proof of concept to investigate the possibility of immune modulation as a treatment for schizophrenia. Neuropsychiatric side effects of prednisolone, such as depression, mania, psychosis, and suicidal ideation, have been reported. These events are dose dependent, as shown in a meta-analysis in which all corticosteroid-induced adverse events reported in all available studies have been described; hazard ratios for all neuropsychiatric side effects, except panic disorder, did not exceed 1.3 for dosages up to 40 mg prednisolone [38]. As prednisolone carries the risk for neuropsychiatric side effects, extra safety rules are necessary when testing its efficacy as augmentation therapy for schizophrenia in the current study [39].

## Methods/design

### Overview

The study procedures described in this paper are comparable to procedures described in a previously published paper by our group related to another randomized clinical trial (RCT) [40]. However, there are essential differences between both studies, such as treatment duration, duration of the trial itself, and an interview used to determine the diagnosis of the participant as well as the intervention.

This study, being a proof of concept trial, was designed as a double-blind placebo-controlled trial to be able to differentiate between clinical effects of prednisolone addition and placebo effects. Prednisolone or placebo is provided in addition to current antipsychotic medication as it is not the intention to replace existing antipsychotic treatments. Finally, by applying the placebo arm and urging treating physicians to keep the antipsychotic dose as stable as possible during the 6-week period, the effect of prednisolone addition can be measured.

In this study, 90 patients with schizophrenia, schizoaffective disorder, schizophreniform disorder, or psychotic disorder not otherwise specified (NOS) will be included; patients are aged 18–70 years and have a time interval between the onset of psychosis and study entry not exceeding 7 years. Patients will continue their antipsychotic medication and (if applicable) other psychotropic drugs throughout the treatment period of 6 weeks. Doses are preferably kept stable, but dose adjustments of up to 25% of the initial dose are allowed. Changes in dosage of antipsychotic medication or benzodiazepines of 25% or more (relative to the baseline visit), will be regarded as a secondary outcome measure.

For this proof of concept study, a treatment period of 6 weeks was considered appropriate. We hypothesize that if prednisolone carries the expected effect, this will be apparent within this period of time. The study does not intend to assess the therapeutic potential of prednisolone as a possible augmentation therapy for these patients. Rather, the results may strengthen the case for immune-modulatory treatment and encourage further research in this direction.

### Aims

This study aims to investigate the effect of prednisolone augmentation to antipsychotic pharmacotherapy in patients with a psychotic disorder. The primary objective of this study will be the change in symptom severity (PANSS) between baseline and end-of-treatment (6 weeks treatment). Secondary objectives include changes in symptom severity in the follow-up phase, cognition, depressive symptoms, global functioning, prediction of treatment response to prednisolone therapy using immune markers (blood samples), and insight into psychosis and inflammation in the brain using magnetic resonance imaging (MRI).

### Recruitment and allocation

#### Recruitment

In total, seven health care centers (academic hospitals and community clinics) will recruit patients for this trial; three Dutch centers, one Belgian center, and three Norwegian centers. Patient clinic lists will be screened at all participating sites for potential patients. Additionally, potential patients will be referred by other healthcare centers and researchers from other trials that are collaborating with the participating centers involved in this trial. In Norway eligible patients will be referred by their treating psychiatrists and physicians.

#### Allocation

All 90 patients will be randomized 1:1 to either prednisolone or placebo for 6 weeks study medication use, in addition to their current antipsychotic medication as prescribed by their treating physician. A randomization list has been created independently by the data management team of the Julius Center, University Medical Center, Utrecht (The Netherlands and Belgium) and by biostatisticians at the University of Bergen, Bergen (Norway). Study medication kits were manufactured based on this randomization list. A web-based application (*https://www.juliuscentrum.nl/random/* in the Netherlands and Belgium) and a paper randomization list are used by blinded study team members to allocate the applicable kit number to each patient. Randomization was stratified for country, center, and gender.

The study staff will not have access to the trial treatment randomization codes. These will be stored in the pharmacy in the University Medical Center Utrecht (The Netherlands), Ziekenhuis Netwerk Antwerpen (Belgium), and Haukeland University Hospital (Norway) in case emergency unblinding is needed. In the case of serious adverse events (SAEs) where knowledge regarding the assigned treatment is important to decide on medical management of the emergency event, unblinding is permitted.

This study is a double-blind placebo controlled trial in which both the patients and the study team members are blind to treatment allocation. None of the study team members have access to the randomization codes. The study physicians who are reviewing laboratory reports for safety purposes are prohibited from collecting any study data for those individual patients. The laboratory results are stored in a location which is not accessible to the study team members conducting protocol procedures.

### Inclusion criteria

1. A DSM-IV-TR diagnosis of 295.x (schizophrenia, schizophreniform disorder, or schizoaffective disorder) or 298.9 (psychosis NOS).

2. Onset of psychosis no longer than 7 years ago.
3. Minimum total PANSS score of 60.
4. Aged 18–70 years.
5. Patients are treated with antipsychotic medication (stable dosage for at least 3 weeks).
6. Written informed consent is obtained.
7. Female patients of childbearing potential need to utilize a proper method of contraception (contraceptive pill, vaginal ring, hormonal patch, intrauterine device, cervical cap, condom, contraceptive injection, diaphragm) in case of sexual intercourse during the study.

Additional inclusion criteria for patients included in Norway are listed in Additional file 1.

### Exclusion criteria

1. Presence of any contraindications of prednisolone as reported in the summary of product characteristics (SPC)
2. Presence of diabetes mellitus or random glucose levels exceeding 11 mmol/L at screening in a non-fasting condition or 7 mmol/L in a fasting condition, severe heart failure, severe osteoporosis, or systemic fungal infections
3. Body mass index (BMI) of > 30.0
4. Current or chronic use of systemic glucocorticosteroids (temporary use is permitted, if stopped 1 month before start of treatment trial)
5. Chronic use of NSAIDs, defined as daily use during more than 2 months; intermittent use is permitted if stopped at least 1 month before start of treatment trial
6. Pregnancy or breast-feeding—a urine pregnancy test will be performed at screening
7. Concurrent use of certain types of medication:
  - Carbamazepine, riphampicine, primidone, barbiturates, and phenytoine
  - HAART medication (both HIV protease inhibitors and (non)-nucleoside reverse transcriptase inhibitors), especially efavirenz, ritonavir, and lopinavir
  - Telaprevir and boceprevir in treatment of hepatitis C

Additional exclusion criteria for patients included in Norway are listed in Additional file 1.

### Interval assessments

In total, 11 visits will be conducted throughout a period of 1 year. The patients are using study medication for 6 weeks and will be followed up for another 46 weeks. The informed consent procedure and the eligibility check will be conducted during the screening visit. When the patient is eligible for study participation, the patient will be randomized to either prednisolone or placebo. During the 6 weeks of study medication intake, the patient will be reviewed weekly by a study physician for adverse events. The symptom severity, which is the primary outcome of this study, will be measured by conducting the commonly used PANSS interview, assessed by a trained researcher. Additionally, the Global Assessment Functioning (GAF) score [41], the Calgary Depression Scale for Schizophrenia (CDS) score [42], drug and alcohol use, and cognition by using the Brief Assessment of Cognition in Schizophrenia (BACS) score [43] will be measured. Blood will be withdrawn at five occasions throughout the study for metabolic and immunological measurements. The patients in Norway will be invited to the optional MRI procedures. An overview of the assessments per visit can be found in Table 1.

**Table 1:** Patient visits and examinations specified per visit

	Visit 1 − 12 to 0 weeks (screening)	Visit 2 week 0 (baseline)	Visit 3 week 1	Visit 4 week 2	Visit 5 week 3	Visit 6 week 4	Visit 7 week 5	Visit 8 week 6 (end of treatment)	Visit 9 week 16	Visit 10 week 26	Visit 11 week 52	Early termination visit*
**Informed consent, MINI, demographics**	X											
**Eligibility criteria**	X											
**Medical history, current medical conditions**	X											
**Physical examination, BMI, ECG**	X											
**Pregnancy testing**	X	X						X				X
MRI**	X										X	
**Use of concomitant medication**	X	X	X	X	X	X	X	X	X	X	X	X
**Dispense study medication**		X	X	X	X	X	X					
**Randomization**		X										
**Drugs/alcohol use**	X				X			X				X
**Side effects, compliance**			X	X	X	X	X	X				X
**PANSS, GAF**	X	X		X		X		X	X	X	X	X
**Suicidal behavior, CDS, hospitalization**		X	X	X	X	X	X	X	X			X
**BACS**		X						X		X		X
**Blood/ urine samples**	X	X	***	***	X	***	***	X		X		X

*MINI* Mini International Neuropsychiatric Interview, *BMI* body mass index, *ECG* electrocardiogram, *MRI* magnetic resonance imaging, *PANSS* Positive And Negative Syndrome Scale, *GAF* General Assessment of Functioning, *CDS* Calgary Depression Scale, *BACS* Brief Assessment of Cognition in Schizophrenia

*Will be performed if a patient prematurely discontinues the study

**Optional part of the study in Norway

***Fasting glucose only, in Norway only

### Training and inter-rater reliability

All involved researchers will be trained in the PANSS interview by an experienced PANSS trainer, using instructional videos and assessment of a test video in a classroom setting or by a training program at the PANSS institute (panss.org). After passing an exam, researchers can perform PANSS ratings for this study. PANSS raters will be assigned to patients in order to limit the bias in PANSS assessment in patients. Proper conduct of The Comprehensive Assessment of Symptoms and History (CASH; the Netherlands and Belgium; demographics, alcohol, and drugs only) interview [44], Mini International Neuropsychiatric Interview 5.0.0 Plus (M.I.N.I. 5.0.0 Plus) [45], and cognitive testing will also be trained by experts. Study team members will be carefully selected and extensively informed and trained regarding Good Clinical Practice (GCP).

### Treatment

All patients included in the study will use the study medication in addition to their current antipsychotic medication as prescribed by their treating physician at the time of inclusion. Study medication kits were prepared by ACE Pharmaceuticals (The Netherlands and Belgium) and Kragerø Tablettproduksjon AS (Norway). They were provided with a randomization list indicating which kit number should contain which treatment. The pharmaceutical company responsible for manufacturing the study medication ensured that the tablets are indistinguishable in appearance, shape, smell, mass, and taste.

Study medication will be tapered-off within 6 weeks in the following schedule: 40 mg/day for 3 days and 30 mg/day for 4 days, followed by a decrease of 5 mg/day per week during the remaining 5 weeks; in the second week, patients will use 25 mg/day, in the third week 20 mg/day, etc. In the last week the patients will only take prednisolone on days 1–3 and day 5 and 7; a tapering scheme in line with the treatment guidelines for inflammatory bowel diseases (2008). Each study medication kit consists of six weekly boxes, each containing all dosages for one week, in which the study medication tablets are sorted according to the tapering-off schedule. Each weekly box is labeled in line with Annex 13 of Clinical Trial Directive.

To prevent loss of bone mass, we will provide calcium supplementation (calciumcarbonate 500 mg daily) and vitamin D supplementation (cholecalciferol 400 IU daily). Pantroprazole will be actively provided to patients with a history of ventricular/duodenal ulcer.

### Outcome variables

#### Primary outcome variables

##### Clinical outcome measure

Our main study parameter is overall symptom severity as measured with the PANSS total score.

#### Secondary outcome variables

##### Symptom severity

Secondary study parameters include PANSS total scores 4, 6, and 12 months after start of the treatment. Furthermore, general functioning will be evaluated using the GAF comparing patients treated with prednisolone versus placebo.

##### Cognitive assessment

Neurocognitive functioning as measured with the BACS will be used to measure the possible improvement over the treatment and the 6-month follow-up period. The BACS was chosen as the neurocognitive measure due to its repeatability as well as the short assessment time (35 min), which is expected to lead to higher completion rates and fewer missing data. The BACS has been developed for assessing the domains of cognitive functioning in schizophrenia trials [43]. It consists of the following domains:

1. Verbal memory: List Learning
2. Working memory: Digit Sequencing Task
3. Motor speed: Token Motor Task
4. Verbal fluency: Category Instances
5. Verbal fluency: Controlled Oral Word Association Test
6. Attention and speed of information processing: Symbol Coding
7. Executive functions: Tower of London

##### Laboratory assessments

Immunological and metabolic parameters will be assessed four times over the 1-year study period. We will investigate inflammatory processes that may be involved in a subpopulation of patients with psychotic disorders using prednisolone augmentation [33]. To investigate immunological biomarkers that predict treatment response to prednisolone therapy, we will collect serum and RNA of all patients at baseline and 3, 6, and 26 weeks after the start of the study medication use. The laboratory assessments will contain the following analyses:

1. Multiplex immunoassay
2. Infectious disease profiling
3. Flow cytometry
4. Selective reaction monitoring (SRM)

##### Brain imaging (optional in Norway)

Information regarding the brain imaging part of the study conducted in Norway can be found in Additional file 2.

#### Other objectives

Severity of depression will be assessed and compared between groups using the CDS. The need to adjust current antipsychotic medication by 25% or more is compared between groups. Finally, safety data will be assessed by comparing incidence (number and percentage of subjects with at least one occurrence) of key SAEs and suspected unexpected serious adverse reactions (SUSARs) between both groups, e.g., hospitalizations.

### Safety assessment

#### Safety measurements

At each visit a study physician (psychiatrist or psychiatry resident) will review the possible adverse events (especially neuropsychiatric side effects), medication use, and any contraindications for prednisolone use. After the screening visit, the blood results will be reviewed by a study physician regarding the inclusion and exclusion criteria. After the baseline visit at which patients start study medication, safety parameters will be checked by a study physician who is not conducting any other protocol assessment. Based on blood assessment, presence of adverse events, and contraindications for prednisolone use, it will be determined if it is still safe to use the study medication.

#### Stopping rules

Several events that may jeopardize the patient’s health will prompt clinicians to end the study and start tapering the patient off the study medication immediately in line with the treatment guidelines for inflammatory bowel diseases (2008). These events include the patient developing:

- A (fasting) blood glucose exceeding 7.0 mmol/L; in case a non-fasting glucose level was determined, a blood glucose level exceeding 11 mmol/L
- Suicidal ideations assessed by the CDS, the following cut-off are applicable based on item 8: Suicidal ideations within the CDS
  - ◦ Score of 0: no action (no suicidal ideation)
  - ◦ Score of 1: consult with clinician (passive suicidal ideation)
  - ◦ Score of 2/3: potential cause to stop study medication treatment—consult with clinician (active suicidal ideation)
- A PANSS positive subscore which increases by 10 or more points without a clear reason (i.e., medication non-adherence)
- PANSS item G6 (depression) exceeding a score of 4
- The need for coercive treatment
- Pregnancy
- Oral systematic infectious disease

#### Adverse events

Any undesirable experience occurring in a subject during the study is defined as an adverse event (AE), regardless of any relatedness to the study medication. All AEs reported spontaneously by the subject or observed by the investigator or study team members conducting the visit will be recorded. AEs will be checked by a study physician every visit by asking an open question about how the patient is doing and if the patient was suffering from any AE. Furthermore, the study physician will assess a standardized questionnaire which includes all potential prednisolone-related AEs. If an AE requires follow-up, the study physician will inform the general practitioner, will examine and monitor the AE him/herself, or, if necessary, will liaise with a medical specialist. In case SAEs have occurred, the applicable authorities will be informed as described in the study protocol following the guidelines. In line with local laws and regulations, participant insurance is in place in case any harm caused by the trial procedures is experienced by the participants.

### Power calculation and statistical analysis

A two group *t*-test with a 0.050 two-sided significance level will have 80% power to detect an effect size of 0.610 when the sample sizes in the two groups are 44 and 44, respectively (a total sample size of 88; text generated by nQuery Advisor). Subsequently, the sample size can be reduced by the data with an Ancova instead of analyzing a *t*-test [46]. The level of reduction depends on the correlation (r) between baseline and follow up measurements: N-ANCOVA = ((1 − r2) × N *t*-test) + 1. From a previous study in this hospital (OPTiMiSE trial) where a PANSS was performed at baseline and after 6 weeks, r = 0.48 was found. Therefore, N-ANCOVA = ((1 − 0.482) × 88) + 1 = 68.7248. Lastly, we expect a considerable number of drop outs. Assuming a drop-out rate of 30%, the definitive sample size is calculated at 68.7248 × 1.3 = 89.34224, which makes a total of *N* = 90 patients.

Statistical analyses for baseline characteristics and the outcomes regarding symptom severity, global functioning, depressive symptoms, and cognition will be performed in IBM SPSS Statistics version 25.0 [47]. A summary of the baseline characteristics will be created in order to assess baseline differences across the treatment groups. Normal distributed data will be expressed as means and standard deviations, and categorical data will be expressed as percentages. The baseline characteristics of the prednisolone and placebo group will be compared and tested with a *t*-test (for continuous variables) and a Chi-square test (for categorical variables). In this study we will use a mixed model analysis. Since we do not estimate a linear change in this analysis, the increase in efficiency compared to the analysis of covariance and therefore the reduction in sample size will not be immense and this calculation will suffice. The mixed model analysis will allow us to use the data of the discontinued patients as it takes into account all observations. This analysis will be used for the objectives regarding symptom severity, global functioning, depressive symptoms, cognition, and MRI. As stratification was performed for gender, center, and country, these variables will be used as covariates in the analyses in addition to variables that are significantly different between treatment groups as well as the unused pills (percentage over the whole treatment period) to take into account compliance with study medication.

Statistical analysis for the prediction of treatment response to prednisolone therapy using immune markers will be conducted using R software. Protein concentrations before and after treatment with prednisolone and placebo will be compared using mixed effects analysis of covariance adjusted for covariates significantly differing between the groups. Association of clinical outcomes with baseline levels of selected biomarkers in the prednisolone treatment group (i.e., prediction of treatment response) will be assessed using fixed effects analysis of covariance with adjustment for significant covariates. Sensitivity analyses will be conducted by excluding covariates from the analysis of covariance models and, if applicable, including outliers.

### Study procedures

#### Screening visit

Potential study participants will be informed regarding all study procedures. They will be provided with an information letter and will be given sufficient time to read all information, ask any questions of the investigator, and liaise with family and friends. Participation in this study is completely voluntarily. When a potential study participant agrees to take part in this study, written informed consent will be obtained by the study physician. After the informed consent procedure has been completed, eligibility criteria will be checked to assess the patient’s eligibility for participation within the study. The M.I.N.I. 5.0.0 Plus will be assessed to confirm the inclusion diagnosis. Additionally, the PANSS and GAF will be assessed, which both relate to the past week. Additionally, several demographical and clinical variables will be assessed, including date of birth, sex, educational level, prior psychiatric disorders, duration of untreated psychosis, and use of drugs and alcohol. The CASH will be used in the Netherlands and Belgium to document the sociodemographic data and drug and alcohol use, whereas similar forms will be used in Norway. Furthermore, the use of concomitant medication, medical history, and current medical conditions will be recorded. The maximum period between screening visit and baseline visit is 12 weeks. If the patient uses co-medication at the screening visit, which is not allowed, it can be washed out during this period. Additionally, physical examination will be conducted by a study physician, including BMI, blood pressure, and temperature. Any abnormality will be discussed with the patient’s treating physician. To rule out pregnancy in female patients, a urine pregnancy test will be performed. Electrocardiogram (ECG) examination will be conducted in all patients above 60 years old, patients with congenital heart defects, and patients with plausible familial heart disorders and patients with a medical history of syncope, cardiac arrythmias, use of antiarrythmic medication, angina pectoris, heart failure, (recent) myocardial infarction, or myocarditis (Cahn et al. 2008). Signs of lipodystrophy will be monitored. Additionally, blood will be drawn to determine and rule out hyperglycemia as well as signs of systemic infection as these are special warnings for the use of prednisolone. Additionally, several blood parameters (blood differentiation, electrolytes, and thyroid, liver, and renal function) will be checked at screening to identify other serious diseases. Patients need to meet all inclusion criteria and none of the exclusion criteria to be regarded as eligible to participate.

#### Baseline visit

At baseline, PANSS and GAF will be administered, in addition to the CDS and the BACS. If the time between screening and baseline is less than 2 weeks, the PANSS will not be repeated at baseline. Additionally, the presence of any exclusion criteria for the current study will be checked as well as suicidal ideation. Subsequently, the patient will be randomized and the study medication will be dispensed for the first time. Patients will be instructed on medication use with regards to contraindicated co-medications. Blood will be drawn to assess immunological parameters. A study letter will be send to the treating psychiatrist, general practitioner, and pharmacist regarding the patient’s participation.

#### Treatment visits

After the baseline visit, patients will be assessed weekly for 6 weeks. During various visits, a trained study team member will interview them using the PANSS, GAF (every other week; visits 2, 4, 6, and 8) and CDS questionnaires (weekly). At each visit, the presence of suicidal ideation is carefully monitored. Co-medication use will be noted as well as side effects and treatment compliance. Except for visit 8, study medication will be dispensed during each visit during the treatment period. Alcohol and drug use will be assessed using the alcohol/drug use section of the CASH during visits 5 and 8 in the Netherlands and Belgium, whereas similar forms will be used in Norway. At visits 2, 5, and 8 blood draws intended for immunological assessments will be performed. After the blood draw the patients need to remain within the hospital (when possible) until the laboratory values are checked by a physician in case follow-up treatment is necessary. Furthermore, pill boxes are returned to the study centers each week to assess compliance with study medication. Cognitive testing will take place at the end (6 weeks) of the treatment period.

#### Follow-up visits

After 16, 26, and 52 weeks (relative to baseline), follow-up visits will take place, during which the PANSS, CDS, and GAF are assessed. Additionally, the current use of medication and adverse events will be noted. At visit 10, blood will be drawn to assess immunological parameters and the BACS will be repeated once more.

#### Patient safety and emergency unblinding

The participants’ treating physicians remain responsible for the day-to-day treatment. Prednisolone has minor mineral-corticosteroid potencies and is able to adequately pass the blood–brain barrier. The side effects and safety profile of prednisolone are well known. The principal investigator will be responsible for the medical and safety concerns related to study participation. Study members can be contacted at the telephone number provided on the emergency card provided the first time the study medication is dispensed and the letters patients receive during the study. This emergency card includes a telephone number from the pharmacy in case emergency unblinding is necessary outside office hours.

Emergency unblinding is only permitted when necessary for the subject’s emergency treatment at the investigator’s discretion or when required by local laws or regulations. Sealed unblinding envelopes, accessible 24/7, are used for emergency unblinding situations. For each medication set, an unblinding envelope is sent to the applicable hospital pharmacy or hospital department, containing the name of the treatment in each specific study medication kit. The pharmacist or representative performing the unblinding will always request details from the study physician regarding patient information and checks whether the unblinding has consequences for the treatment of the adverse events causing the emergency unblinding.

#### Patient withdrawal

Subjects can quit study participation at any time for any reason without any consequences if they wish to do so. The investigator can decide to withdraw a subject from the study for urgent medical reasons.

Reasons to terminate a patient’s participation include but are not limited to the following:

- The patient withdraws her/his consent
- Intolerance to the study medication
- Start of the use of NSAIDs, HAART, telaprevir, boceprevir, riphamicine, primidone, barbiturates, and phenytoine
- Administration of a live vaccine is needed
- Any of the stopping rules provided in section 2.10

In case a patient or the investigator decides to terminate a patient’s participation, the patient needs to be followed until the patient has completely tapered-off the study medication. This tapering-off scheme will be decided by a study physician based on the patient’s situation. If needed, the study physician will liaise with a medical specialist (e.g., endocrinologist).

#### Incentives

The patients will be compensated for the time and effort related to their participation in this trial. Patients in The Netherlands and Belgium will receive 25 euros per completed study visit and all travel expenses will be reimbursed. Patients in Norway will receive 200 Norwegian kroners for their time investment and travel expenses.

### Data collection, management, and dissemination of the results

Data will be collected on paper during the study visits and will be inserted in an electronic case report form (eCRF). The paper documentation will be stored in a locked cabinet, accessible for authorized personnel only. Data collected during visits with study participants will be pseudonymized and will be treated as confidential. The link between the patient identifiers and the subject study number is documented on a subject identification code list, which is only accessible for authorized study team members.

Once all data have been inserted in the eCRF and verified by the monitor, the data will be signed off by the principal investigator. Hereafter the eCRF will be locked, after which the data in the eCRF cannot be changed. Data managers will create the final database. The final anonymized database will be shared with the Stanley Medical Research Institute for the data obtained in the Netherlands and Belgium. Specific data management procedures can be provided by the trial coordinator on request.

The results of this study will be presented as abstracts, oral presentations, or posters, as well as publication in an international peer-reviewed journal. Patients who requested to be informed regarding the outcome of the trial will receive an information letter containing the overall outcome of the study.

### Ethical and regulatory standards

#### Medical ethics review board

Ethics approval was obtained from the research and ethics committee of the University Medical Center Utrecht (UMCU), the Netherlands, protocol number 14–110, Ziekenhuis Netwerk Antwerpen (ZNA), Belgium and Haukeland University Hospital, Norway, protocol number 2017/620. Approval was also obtained from the Norwegian Medicines Agency. The trial is registered in the ClinicalTrails.gov database (identifier NCT02949232 for the Netherlands and Belgium and NCT03340909 for Norway) and the European Clinical Trials Database (EudraCT number 2014–000520-14 for the Netherlands and Belgium and 2017–000163-32 for Norway).

#### Data and safety monitoring board

The safety of the study will be judged by an independent committee of experts (data and safety monitoring board) on a regular basis, at a frequency of at least once a year. The members of this board will have access to all safety and progress information (e.g., inclusion and drop-out rates). If deemed necessary, the DSMB members may review the unblinded study data. The DSMB will meet each time after ten additional patients are recruited, but meetings may be more frequent depending on trial events causing safety concerns, the enrolment rate (much slower or faster than anticipated), or when the DSMB deems this to be appropriate for other reasons. The DSMB may suggest changes to the protocol or provide an altered judgment of feasibility if information from the annual safety report or new information about the study medication becomes available. No interim analyses are planned. The tasks and responsibilities of the DSMB, as well as premature termination criteria, are described in a separate DSMB charter, which can be provided by the trial coordinator on request.

#### Declaration of Helsinki

The study will be conducted in accordance with this protocol as well as the principles of the Declaration of Helsinki (64th WMA general assembly; Fortaleza, Brazil, October 2013), the ICH-GCP guidelines, and other applicable laws and regulations.

## Discussion

This study was developed to explore pharmacotherapy options with an anti-inflammatory agent to improve both clinical symptoms and cognition in patients with a psychotic disorder. Prednisolone was chosen due to its potent, broad acting immune modulating working mechanism. The potential positive effect of prednisolone augmentation to current antipsychotic medication use will be investigated in this study. Given its potential to reduce microglia activity in the brain and lower activity of both the innate and the acquired immune system, it is expected that prednisolone will lower the symptom severity (decrease in PANSS total and subscores) and will improve cognition (especially attention, executive functioning, and verbal memory) and global functioning in comparison to placebo.

If we are to find efficacy of prednisolone addition, a second step is to investigate exactly which immune components mediate this effect. For this purpose, blood is drawn at regular intervals to monitor baseline values and changes in inflammatory blood markers. Positive effects on symptoms and cognition have been demonstrated from corticosteroids used to treat MS, traumatic brain injury, and stroke, disorders in which an immune component has clearly been described [48]. A negative finding in the current study might mitigate our enthusiasm to test other anti-inflammatory drugs as augmentation for treatment options. Corticosteroids have been described to elicit psychiatric symptoms. Therefore, medication is given in a moderate dose and medical assessments are frequently performed to screen for suicidality and other safety issues. We expect that the results of this study, be it positive or negative, will give strong direction to the emerging field of immune-psychiatry for the treatment of psychosis.

## Trial status

The study is active and currently recruiting patients. Recruitment started in March 2015. The recruitment in The Netherlands and Belgium was completed end of 2018 and the final assessments were performed in 2019. Recruitment in Norway will be completed by the end of 2021. The final assessments in Norway will be conducted at the end of 2022. The following protocol versions are currently used; protocol version 9.0 dated 19 December 2018 in the Netherlands, protocol version 8.0 dated 28 September 2018 in Belgium, and protocol version 6.0 dated 04 January 2019 in Norway.

## Supplementary information

**Additional file 1.** Additional eligibility criteria for patients included in Norway.

**Additional file 2.** Brain imaging (optional part of the study in Norway).

## Data Availability

Not applicable.

## Abbreviations

BACS: Brief Assessment of Cognition in Schizophrenia
CASH: Comprehensive Assessment of Symptoms and History
CDS: Calgary Depression Scale
CNS: Central nervous system
CSF: Cerebrospinal fluid
COX-2: Cycloxygenase-2
DSM –IV: Diagnostic and Statistical Manual of Mental Disorders-IV
GAF: Global Assessment of Functioning
GM: Grey matter
GWAS: Genome-wide association study
MHC: Major histocompatibility complex
M.I.N.I. 5.0.0 Plus: Mini International Neuropsychiatric Interview 5.0.0 Plus
MRI: Magnetic resonance imaging
NSAIDs: Non-steroidal anti-inflammatory drugs
PANSS: Positive and Negative Syndrome Scale
QoL: Quality of life
SAE: Serious adverse event
SUSAR: Suspected Unexpected Serious Adverse Reaction
WM: White matter
