# Supplementary material for: Prednisolone versus placebo addition in the treatment of patients with recent-onset psychotic disorder: a trial design
Source: Trials. 2020 Jun 8;21:492. doi: 10.1186/s13063-020-04365-4 (PMC7278136; doi:10.1186/s13063-020-04365-4)
Supplement: Supplementary file 1 — Additional file 1. Additional eligibility criteria for patients included in Norway. [file 13063_2020_4365_MOESM1_ESM.docx]

Additional file 1: Additional eligibility criteria for patients included in Norway

Additional exclusion criteria:

1. Presence of any of the contra-indications of prednisolone as reported in the SPC. These include hypersensitivity to any ingredients in the formulation, systemic infections unless specific anti-infective therapy is employed, patients with ocular herpes simplex due to the possibility of perforation, recent vaccination with live or weakened virus or bacteria. Also the following special warnings in the SPC will represent exclusion criteria: Existing or previous history of severe affective disorders in themselves or in their first degree relatives, including depressive or bipolar disorders or previous steroid psychosis, glaucoma or family history of glaucoma, hypertension or heart failure, liver impairment and/ or failure, epilepsy, osteoporosis, peptic ulceration, previous steroid myopathy, renal insufficiency, history of tuberculosis or x-ray changes characteristic of tuberculosis, recent myocardial infarction, chickenpox, measles.
2. Exclusion criteria for the optional MRI procedure (not exclusion criteria for participation in the rest of the study):
   1. Pacemaker
   2. Aneurism clips*
   3. Neuro stimulator*
   4. Foreign metallic object in the eye
   5. Ear implant*
   6. V/P shunt*
   7. Coronary intervention/ operation*
   8. Claustrophobia
   9. Other metal implant/ prosthesis*
   10. *To be resolved on an individual basis based on discussion with the Radiology department.
