# Supplementary material for: Prednisolone versus placebo addition in the treatment of patients with recent-onset psychotic disorder: a trial design
Source: Trials. 2020 Jun 8;21:492. doi: 10.1186/s13063-020-04365-4 (PMC7278136; doi:10.1186/s13063-020-04365-4)
Supplement: Supplementary file 2 — Additional file 2. Brain imaging (optional part of the study in Norway). [file 13063_2020_4365_MOESM2_ESM.docx]

Additional file 2: Brain imaging (optional part of the study in Norway)

Structural and functional magnetic resonance imaging (MRI/ fMRI) will be conducted at baseline, and then between weeks 6 and 8 thereafter, and finally after 12 months. The exploratory analyses are expected to shed light on how psychosis and inflammation, as well as anti-inflammatory treatment influences the brain at the structural and functional levels at both the group and individual level, and the relationship to peripheral inflammatory markers.

The following techniques will be applied during the same session (with relevant targets/ outcome measures in parentheses):

1. Structural (anatomical) MRI (whole brain, WM and GM volumes)

2. BOLD fMRI (functional properties using the blood-oxygen-level dependent (BOLD) contrast to assess neuronal activity)

3. MRI spectroscopy (levels of neurotransmitters and markers of neuronal stress among others)

4. Diffusion tensor imaging (DTI) (investigates WM integrity)

5. Free water imaging (a subtype of DTI that investigates micro-edema of the brain, supposedly a reflection of brain inflammation)
